# Prognostic factors in patients with high-risk stage II colon cancer after curative resection: a post hoc analysis of the JFMC46-1201 trial

**DOI:** 10.1007/s00384-023-04559-7

**Published:** 2023-10-31

**Authors:** Kiichi Sugimoto, Kazuhiro Sakamoto, Takashi Tsuchiya, Takao Takahashi, Hiroki Ohge, Toshihiko Sato, Masataka Taguri, Satoshi Morita, Sotaro Sadahiro

**Affiliations:** 1 Department of Coloproctological Surgery, Juntendo University, 2-1-1 Hongo, Bunkyo-ku, Tokyo 113-8421 Japan; 2 Department of Surgery, Sendai City Medical Center, 5-22-1 Tsurugaya, Miyagino-ku, Sendai, Miyagi 983-0824 Japan; 3 Department of Digestive Surgery, Gifu University Hospital, 1-1 Yanagido, Gifu, 501-1194 Japan; 4 Department of Infectious Diseases, Hiroshima University Hospital, 1-2-3 Kasumi, Minami-ku, Hiroshima, 734-8551 Japan; 5 Department of Surgery, Yamagata Prefectural Central Hospital, 1800 Aoyagi, Yamagata, 990-2292 Japan; 6 Department of Health Data Science, Tokyo Medical University, 6-1-1 Shinjuku, Shinju-ku, Tokyo 160-8402 Japan; 7 Department of Biomedical Statistics and Bioinformatics, Kyoto University, 54 Kawahara-cho, Shogoin, Sakyo-ku, Kyoto, 606-8507 Japan; 8 Department of Surgery, Tokai University, 143 Shimokasuya, Isehara, Kanagawa 259-1193 Japan

**Keywords:** Colon cancer, High-risk stage II, Postoperative adjuvant chemotherapy, Uracil and tegafur plus leucovorin (UFT/LV), CEA mRNA

## Abstract

**Purpose:**

The goal of the current study was to identify prognostic factors for disease-free survival (DFS) and overall survival (OS) in high-risk stage II colon cancer.

**Methods:**

The subjects were patients with histologically confirmed stage II colon cancer undergoing R0 resection who met at least one of the following criteria: T4, perforation/penetration, poorly differentiated adenocarcinoma, mucinous carcinoma, and < 12 examined lymph nodes. Patients self-selected surgery alone or a 6-month oral uracil and tegafur plus leucovorin (UFT/LV) regimen. Serum CEA mRNA at ≥ 24 h after surgery and < 2 weeks after registration was also examined as a potential prognostic factor for stage II colon cancer. This study is registered with UMIN-CTR (protocol ID: UMIN000007783).

**Results:**

1880 were included in the analysis to identify prognostic factors for DFS and OS in patients with high-risk stage II colon cancer. In multivariate analyses, gender, depth of tumor invasion, extent of lymph node dissection, number of examined lymph nodes, and postoperative adjuvant chemotherapy (POAC) emerged as significant independent prognostic factors for DFS. Similarly, multivariate analysis showed that age, gender, depth of tumor invasion, perforation/penetration, extent of lymph node dissection, number of examined lymph nodes, and POAC were significant independent prognostic factors for OS. Univariate analyses showed no significant difference in DFS or OS for CEA mRNA-positive and mRNA-negative cases.

**Conclusion:**

This study showed that gender, depth of tumor invasion, extent of lymph node dissection, number of examined lymph nodes, and lack of use of POAC were significant independent prognostic factors in stage II colon cancer.

## Introduction

Colorectal cancer (CRC) is the third most common malignancy and the second most frequent cause of cancer-related mortality worldwide [1]. In the absence of conclusive randomized controlled trial data, clinical guidelines published by the American Society of Clinical Oncology (ASCO), the National Comprehensive Cancer Network (NCCN), and the European Society for Medical Oncology (ESMO) have suggested a benefit of postoperative adjuvant chemotherapy (POAC) in stage II colon cancer with risk factors for recurrence [2–5]. Clinical guidelines from the Japanese Society for Cancer of the Colon and Rectum also recommend POAC for stage II CRC with risk factors for recurrence, but not for cases without these risk factors [6]. However, such risk factors have yet to be clearly established [7].

We previously performed a prospective observational study (JFMC46-1201) to judge the efficacy of POAC with oral uracil and tegafur plus leucovorin (UFT/LV) for stage II colon cancer with potential risk factors for recurrence [8]. Here, we describe a post hoc analysis of this study, with the goal of identifying prognostic factors for disease-free survival (DFS) and overall survival (OS). In addition to prevalent clinicopathological factors, serum positivity for carcinoembryonic antigen (CEA) mRNA has been proposed as a surrogate marker for circulating tumor cells (CTCs) [9–12], and the presence of CTCs in peripheral blood at ≥ 24 h after surgery has been reported to be an independent risk factor [13]. Therefore, in the current study, serum CEA mRNA at ≥ 24 h after surgery and < 2 weeks after registration was also examined as a potential prognostic factor for stage II colon cancer.

## Materials and methods

### Patients

Details of the study protocol have been reported elsewhere [7]. The subjects were patients with histologically confirmed stage II colon cancer undergoing R0 resection who met at least one of the following criteria: T4, perforation/penetration, poorly differentiated adenocarcinoma, mucinous carcinoma, and < 12 examined lymph nodes. The patients were aged 20–80 years and had ECOG PS of 0 or 1. Patients were registered after the confirmation of pathological findings. Patients self-selected surgery alone (Group A) or a 6-month oral UFT/LV regimen (Group B). The study was performed in accordance with the ethical standards of the 1975 Declaration of Helsinki and its later amendments. This study is registered with UMIN-CTR (protocol ID: UMIN000007783).

### Clinicopathological analysis

The following clinicopathological factors were analyzed: age, gender, location (right-side/left-side), depth of tumor invasion (TNM classification [14]), presence of perforation/penetration, histology of the primary tumor [15], extent of lymph node dissection [15], number of examined lymph nodes, number of patients at each center, and use of POAC. D3 lymph node dissection means the complete removal of the pericolic, intermediate, and main lymph nodes with a high vascular tie at the root of the feeding artery. D2 lymph node dissection means the complete removal of the pericolic and intermediate lymph nodes without a high vascular tie. D1 lymph node dissection means the complete removal of the pericolic lymph nodes. D0 lymph node dissection means the incomplete removal of the pericolic lymph nodes. Since the JFMC46-1201 prospective observational study was a multicenter trial, the evaluation of lymphatic or vascular or perineural invasion may have varied [16], and thus, these factors were excluded from the study. Microsatellite instability (MSI) was also excluded because evaluation of MSI status was not covered by Japanese health insurance during the study period.

### Carcinoembryonic antigen (CEA) mRNA in peripheral blood

Peripheral blood samples were collected at ≥ 24 h after surgery and < 2 weeks after registration. To prevent contamination with epithelial cells, the samples were obtained through a catheter inserted into the peripheral vessel and the first 10 ml of blood was discarded. As described elsewhere [9], total RNA was isolated from 7 ml of peripheral blood using an RNeasy Mini Kit (Qiagen, Germantown, MD, USA) and redissolved in diethyl pyrocarbonate-treated water. 1 μg of isolated total RNA was reverse-transcribed into complementary DNA (cDNA) using Random primer (Sigma-Aldrich, Darmstadt, Germany) and an M-MLV Reverse Transcriptase (Thermo Fisher Scientific, Waltham, MA, USA). Each quantitative RT-PCR (qRT-PCR) contained 2.5 µl of cDNA product, 2.5 µl of PCR buffer, 2.5 µl of deoxynucleotide triphosphate (dNTP), 3.5 µl of 25 mM MgCl_2_, 1.25 µl of forward and reverse primers, 1.25 µl of probe and 0.125 µl of Taq Gold probe (Thermo Fisher Scientific, Waltham, MA, USA), made up to a total volume of 25 µl with sterile water. qRT-PCR was performed using a 10 µM forward primer, 10 µM reverse primer and 2 µM probes, with cycling conditions of 95 °C for 10 min, followed by 53 cycles of 95 °C for 25 s, 65 °C for 1 min, and 72 °C for 30 s. The forward and reverse primers and probe (Human qRT-PCR step for CEA No. 4391675-T, Nihon Gene Research Laboratory Inc., Miyagi, Japan) are synthesized to yield a band of 135 bp of *CEACAM5* (Nt.1811–1945). The CEA mRNA was analyzed by performance of the GAPDH competitive PCR as an internal control. Primers for GAPDH were as follows: forward: 5′-TGAAGGTCGGAGTCAACGG-3′ and reverse: 5′-AGAGTTAAAAGCAGC CCTGGTG-3′; probe for GAPDH is as follows: 5′-TTTGGTCGTATTGGGCGCCTGG-3′. The CEA mRNA/GAPDH mRNA ratio was used to determine the relative CEA mRNA expression level. In the current study, more than 50 copies/μg of the CEA mRNA/GAPDH mRNA ratio was determined as positive.

### Treatment and surveillance

In Group A, postoperative follow-up procedures included clinical assessment and serum CEA measurements every 3 months and chest and abdominal CT every 6 months until 5 years postoperatively or until recurrence, another malignancy or death. Colonoscopy was performed at one and three years after the operation. In Group B, UFT/LV was started within 8 weeks after surgery, with five courses given as UFT (300 mg/m^2^/day) plus LV (75 mg/day) administered orally in 3 doses per day at approximately 8-h intervals. Treatment was administered daily for 28 days followed by a 7-day rest period (daily treatment regimen) or daily for 5 days followed by a 2-day rest period (5-day treatment plus 2-day rest regimen; no treatment on Saturdays and Sundays). For both regimens, one course lasted 5 weeks, and 5 courses were given. After completion of UFT/LV therapy, patients were followed up using the same schedule as that for Group A.

### Statistical analysis

In the current study, DFS is defined as survival from registration for colon cancer until first recurrence of the disease or secondary cancer or mortality from any cause. OS is defined as survival from registration for colon cancer until mortality from any cause. Multivariate analysis was performed using Cox proportional hazard regression by backward elimination method from prognostic factors (age, gender, location, depth of tumor invasion, perforation/penetration, poorly differentiated component, mucinous component, number of examined lymph node, number of participating patients at each institution, and POAC) with an exclusion criterion of *p* = 0.05. All analyses were performed using SAS version 9.4 (SAS Institute Inc., Cary, NC, USA), with differences considered significant at *p* < 0.05.

## Results

### Patient characteristics

**Fig. 1:**
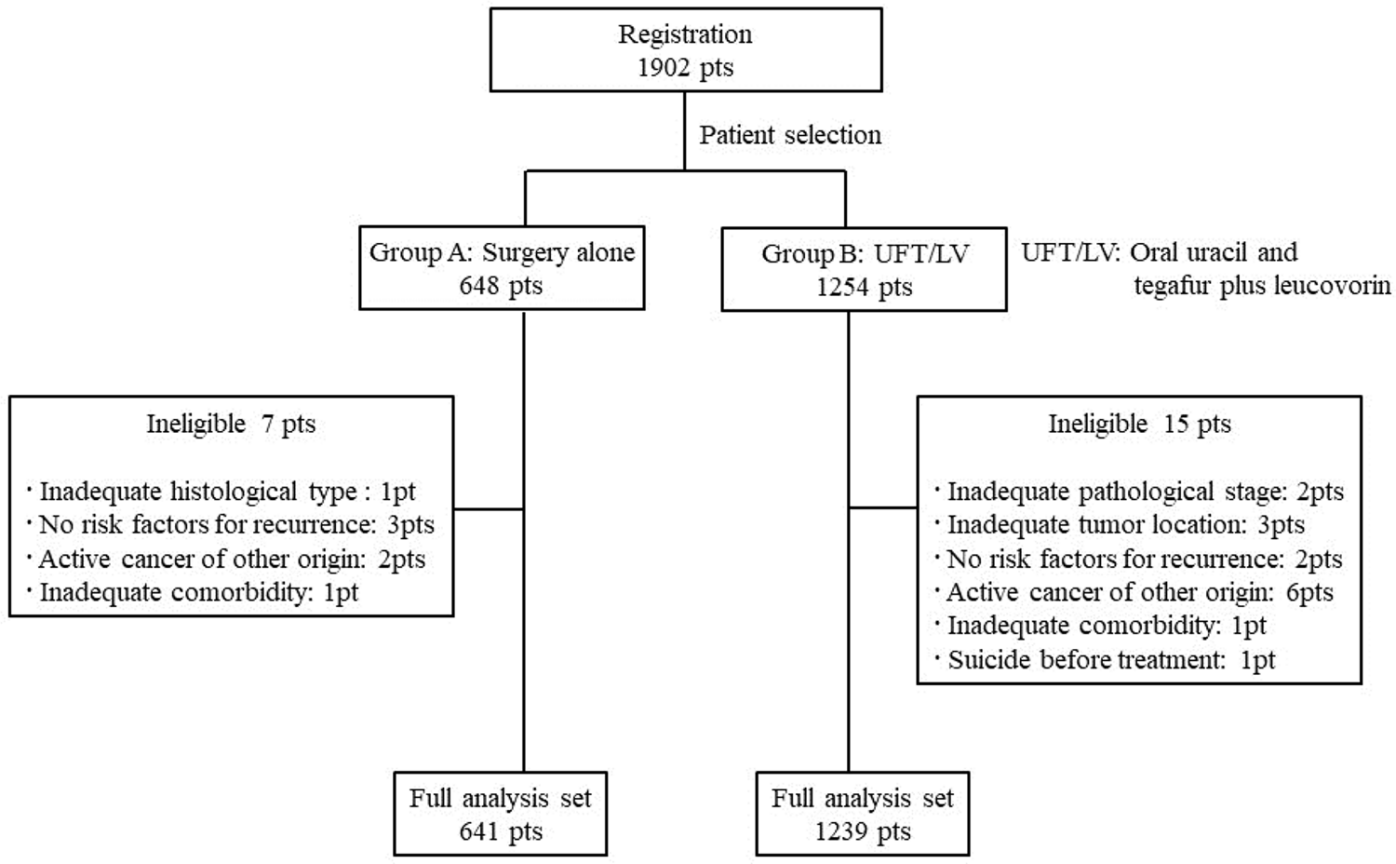
Study scheme. 1902 were included as subjects in the current study. There were 648 patients in Group A (surgery alone) and 1254 patients in Group B (UFT/LV treatment). Among the 1902 patients, 1880 were included in the analysis to identify prognostic factors for DFS and OS in patients with high-risk stage II colon cancer after curative resection

A total of 1938 patients were enrolled in the JFMC46-1201 study at 321 institutions in Japan between May 2012 and April 2016 [8]. Of these patients, 1902 in the non-randomized arm were included as subjects in the current study (Fig. 1). There were 648 patients in Group A (surgery alone) and 1254 patients in Group B (UFT/LV treatment). Among the 1902 patients, 1880 were included in the analysis to identify prognostic factors for DFS and OS in patients with high-risk stage II colon cancer after curative resection. The median follow-up period was 59 months. The baseline characteristics of the 1880 eligible patients are summarized in Table 1. The mean age was 66.7 ± 9.6 years, the median age was 68 (range 29–80) years, and 845 patients (44.9%) were ≥ 70 years old. There were 1023 males (54.4%) and 857 females (45.6%). Tumor locations were the right-side colon (*n* = 803 cases, 42.7%) and the left-side colon (*n* = 1077, 57.3%); the surgical approach was laparotomy (*n* = 801, 42.6%) and laparoscopy (*n* = 1079, 57.4%); the most frequent histological type was moderately differentiated adenocarcinoma (*n* = 890, 47.3%), followed by well-differentiated adenocarcinoma (*n* = 538, 28.6%); the depth of tumor invasion was T3 (*n* = 858, 45.6%) and T4 (*n* = 1022, 54.4%); the extent of lymph node dissection was D3 (*n* = 1598, 85.0%), D2 (*n* = 271, 14.4%), D1 (*n* = 10, 0.5%), and D0 (*n* = 1, 0.1%), and the mean and median numbers of examined lymph nodes were 20 and 17, respectively, with a range of 1–129. The criteria for enrollment were T4 (*n* = 1022, 54.4%), perforation/penetration (*n* = 200, 10.6%), poorly differentiated adenocarcinoma (*n* = 173, 9.2%), mucinous adenocarcinoma (*n* = 262, 13.9%), and < 12 examined lymph nodes (*n* = 701, 37.3%).

**Table 1:** Patient characteristics

Clinicopathological factors	Variables	*n*	(%)
Age	Mean (± SD) Median (range)	66.7 68.0	(± 9.6) (29–80)
Age	< 70 ≥ 70	1035 845	(55.1%) (44.9%)
Gender	Male Female	1023 857	(54.4%) (45.6%)
Location of colon cancer	Right-sided (cecum, ascending, transverse) Left-sided (descending, sigmoid, rectosigmoid)	803 1077	(42.7%) (57.3%)
Surgical approach	Laparotomy Laparoscopy	801 1079	(42.6%) (57.4%)
Histological type	Papillary adenocarcinoma Well differentiated adenocarcinoma Moderately differentiated adenocarcinoma Solid poorly differentiated adenocarcinoma Non-Solid poorly differentiated adenocarcinoma Mucinous adenocarcinoma Signet-ring cell carcinoma	25 538 890 131 38 256 2	(1.3%) (28.6%) (47.3%) (7.0%) (2.0%) (13.6%) (0.1%)
Depth of tumor invasion (TNM classification)	T3 T4	858 1022	(45.6%) (54.4%)
Extent of lymph node dissection^a^	D3 D2 D1 D0	1598 271 10 1	(85.0%) (14.4%) (0.5%) (0.1%)
Number of examined lymph nodes	Mean (± SD) Median (range)	20.0 17.0	(± 14.0) (1–129)
Criteria for enrollment^b^	T4 Perforation/penetration Poorly differentiated adenocarcinoma Mucinous adenocarcinoma < 12 examined lymph nodes	1022 200 173 262 701	(54.4%) (10.6%) (9.2%) (13.9%) (37.3%)

^a^D3: complete removal of pericolic, intermediate, and main lymph nodes with a high vascular tie at the root of the feeding artery. D2: complete removal of pericolic and intermediate lymph nodes without a high vascular tie. D1: complete removal of pericolic lymph nodes. D0: Incomplete removal of pericolic lymph nodes

^b^Duplication was involved

### Events for disease-free survival (DFS) and overall survival (OS)

There were 475 events (25.3%) associated with DFS, including recurrence in 303 cases (16.1%), secondary cancer in 145 (7.7%), and mortality in 27 (1.4%) (Table 2). The most frequent recurrence site was the liver (*n* = 103, 5.5%), followed by the peritoneum (*n* = 85, 4.5%); the most frequent site of secondary cancer was the lung/bronchus (*n* = 29, 1.5%), followed by the colorectum (*n* = 21, 1.1%); and the most frequent cause of mortality was pneumonia (*n* = 8, 0.4%) (Table 2). Regarding OS, there were 160 deaths (Table 3). The most frequent cause of mortality was primary cancer (*n* = 104, 5.5%), followed by cancer of other origin (*n* = 22, 1.2%).

**Table 2:** Details of the events of recurrence/secondary cancer/mortality for disease-free survival (DFS)

Recurrence		Secondary cancer		Mortality	
Site	*n*^a^ (%)	Site	*n*^a^ (%)	Cause	*n* (%)
Liver	103 (5.5%)	Lung / bronchus	29 (1.5%)	Pneumonia	8 (0.4%)
Lung	72 (3.8%)	Colorectum	21 (1.1%)	Cerebrovascular disease	3 (0.2%)
Peritoneum	85 (4.5%)	Stomach	18 (1.0%)	Cardiovascular disease	2 (0.1%)
Anastomotic site	26 (1.4%)	Prostate	12 (0.6%)	Primary cancer	1 (0.1%)
Local	25 (1.3%)	Breast	12 (0.6%)	Senility	1 (0.1%)
Distant lymph node	20 (1.1%)	Liver	9 (0.5%)	Others	6 (0.3%)
Ovary	8 (0.4%)	Pancreas	4 (0.2%)	Unknown	6 (0.3%)
Regional lymph node	6 (0.3%)	Uterus	3 (0.2%)		
Uterus	2 (0.1%)	Gall bladder/bile duct	2 (0.1%)		
Others	19 (1.0%)	Others	40 (2.1%)		
Total	303 (16.1%)	Total	145 (7.7%)	Total	27 (1.4%)

^a^Duplication was involved

**Table 3:** Details of the events of mortality for overall survival (OS)

Cause of mortality	*n*	(%)
Primary cancer	104	(5.5%)
Cancer of other origin	22	(1.2%)
Pneumonia	10	(0.5%)
Cardiovascular disease	3	(0.2%)
Cerebrovascular disease	3	(0.2%)
Senility	1	(0.1%)
Others	8	(0.4%)
Unknown	9	(0.5%)
Patients with mortality	160	(8.5%)

### Prognostic factors for DFS and OS

In multivariate analyses, gender (male) (HR: 1.29 [1.07–1.55]; *p* = 0.0064), depth of tumor invasion (T4) (HR: 2.27 [1.82–2.83]; *p* < 0.0001), extent of lymph node dissection (D0-2) (HR: 1.29 [1.01–1.65]; *p* = 0.0391), number of examined lymph nodes (< 12) (HR: 1.76 [1.41–2.19]; *p* < 0.0001), and POAC (absent) (HR: 1.47 [1.22–1.77]; *p* < 0.0001) emerged as significant independent prognostic factors for DFS (Table 4). No other clinicopathological factors had an independent association with DFS. Similarly, multivariate analysis showed that age (≥ 70 years) (HR: 1.42 [1.03–1.96]; *p* = 0.0318), gender (male) (HR: 1.71 [1.23–2.37]; *p* = 0.0014), depth of tumor invasion (T4) (HR: 3.22 [2.21–4.70]; *p* < 0.0001), perforation/penetration (present) (HR: 1.61 [1.03–2.53]; *p* = 0.0372), extent of lymph node dissection (D0-2) (HR: 1.51 [1.01–2.25]; *p* = 0.0432), number of examined lymph nodes (< 12) (HR: 2.35 [1.63–3.39]; *p* < 0.0001), and POAC (absent) (HR: 1.56 [1.13–2.15]; *p* = 0.0073) were significant independent prognostic factors for OS (Table 4). No other clinicopathological factors showed a significant association with OS.

**Table 4:** Prognostic factors for disease-free survival (DFS) and overall survival (OS)

Clinicopathological factors^a^	Variables	Disease-free survival (DFS)			Overall survival (OS)		
		HR	95% CI	*p*-value	HR	95% CI	*p*-value
Age	< 70				1		0.0318
	≥ 70				1.42	1.03–1.96	
Gender	Female	1		0.0064	1		0.0014
	Male	1.29	1.07–1.55		1.71	1.23–2.37	
Location	Right-sided						
	Left-sided						
Depth of tumor invasion (TNM classification)	T3	1		< 0.0001	1		< 0.0001
	T4	2.27	1.82–2.83		3.22	2.21–4.70	
Perforation/penetration	Absent				1		0.0372
	Present				1.61	1.03–2.53	
Poorly differentiated component	Absent						
	Present						
Mucinous component	Absent						
	Present						
Extent of lymph node dissection^b^	D3	1		0.0391	1		0.0432
	D0-2	1.29	1.01–1.65		1.51	1.01–2.25	
Number of examined lymph node	≥ 12	1		< 0.0001	1	1.63–3.39	< 0.0001
	< 12	1.76	1.41–2.19		2.35		
Number of participating patients at each institution	≥ 5						
	< 5						
Postoperative adjuvant chemotherapy	Present	1		< 0.0001	1		0.0073
	Absent	1.47	1.22–1.77		1.56	1.13–2.15	

^a^Prognostic factors were selected by multivariate analysis with an exclusion criterion of *p* = 0.05

^b^D3: complete removal of pericolic, intermediate, and main lymph nodes with a high vascular tie at the root of the feeding artery. D2: complete removal of pericolic and intermediate lymph nodes without a high vascular tie. D1: complete removal of pericolic lymph nodes. D0: incomplete removal of pericolic lymph nodes

### Clinical impact of CEA mRNA in peripheral blood on DFS and OS

**Fig. 2:**
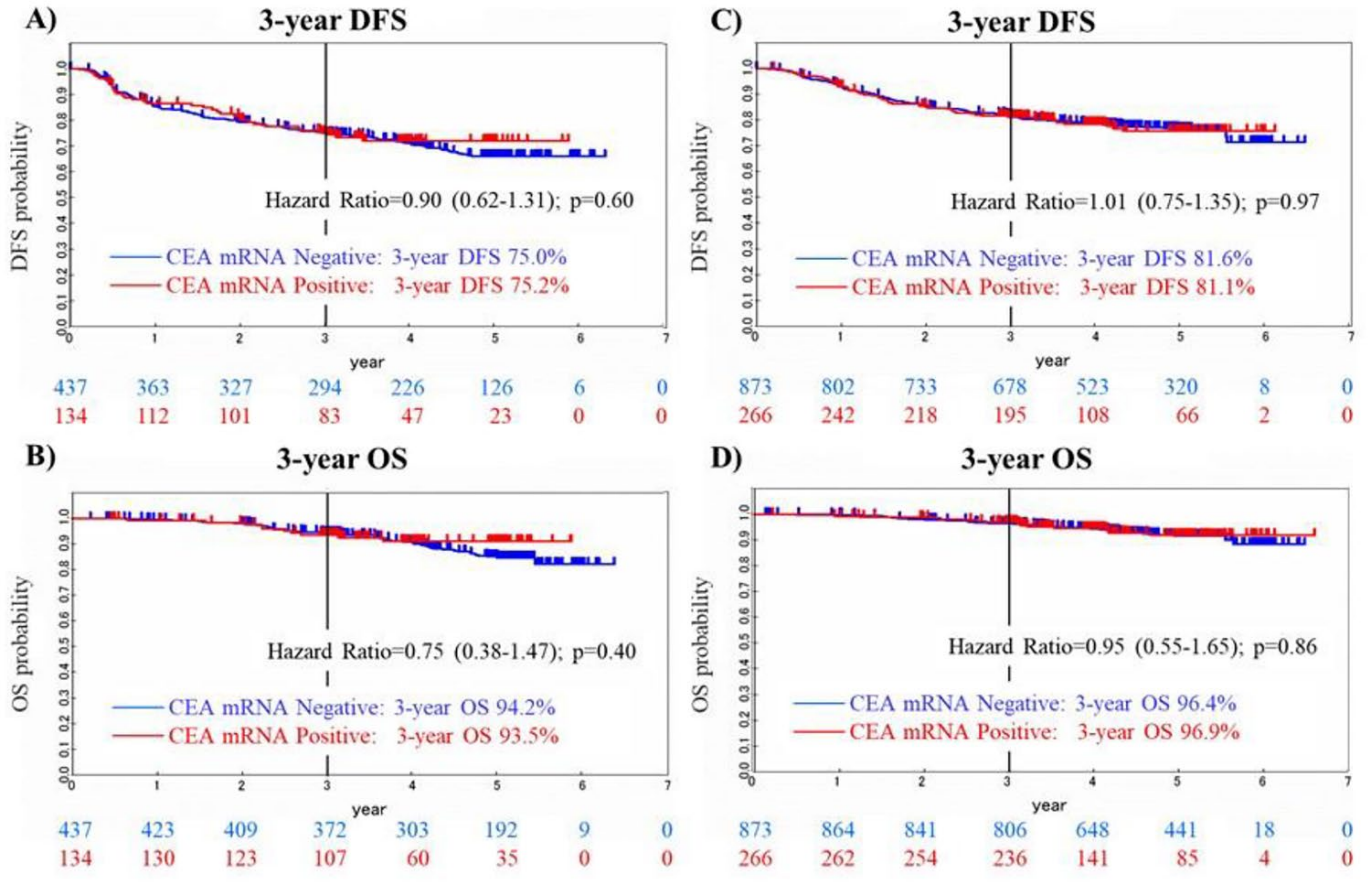
Clinical impact of CEA mRNA in peripheral blood on DFS and OS. CEA mRNA in peripheral blood was measured in 1710 patients, and 400 (23.4%) were found to be positive for CEA mRNA. Univariate analyses showed no significant difference in 3-year DFS or 3-year OS for CEA mRNA-positive and -negative cases in group A (DFS: HR = 0.90 (0.62–1.31); *p* = 0.60; OS: HR = 0.75 (0.38–1.47); *p* = 0.40) (**A**, **B**) or in group B (DFS: HR = 1.01 (0.75–1.35); *p* = 0.97, OS: HR = 0.95 (0.55–1.65); *p* = 0.86) (**C**, **D**)

CEA mRNA in peripheral blood was measured in 1710 patients, and 400 (23.4%) were found to be positive for CEA mRNA. Univariate analyses showed no significant difference in 3-year DFS or 3-year OS for CEA mRNA-positive and mRNA-negative cases in Group A (DFS: HR = 0.90 (0.62–1.31); *p* = 0.60; OS: HR = 0.75 (0.38–1.47); *p* = 0.40) (Fig. 2A, B) or in Group B (DFS: HR = 1.01 (0.75–1.35); *p* = 0.97, OS: HR = 0.95 (0.55–1.65); *p* = 0.86) (Fig. 2C, D).

## Discussion

Among patients diagnosed with adenocarcinoma of the colon, 25–40% have stage II disease, and these cases have a relatively good prognosis, with 5-year OS rates of 72–85% [17] compared with stage III cases [18, 19]. Therefore, there appears only to be a small benefit of POAC, but this may be more important in cases with high-risk features [20]. This indicates the importance of identification of patients who may benefit from POAC [21]. Many studies have examined methods for better identification and treatment of stage II cases with a risk of recurrence, but it has been difficult to find reliable prognostic factors [20]. This makes risk stratification difficult, which causes physicians to individualize treatment and produces widely varied clinical practice [22]. For these reasons, we examined prognostic factors in stage II colon cancer in the cohort from our previous prospective clinical study.

The current study showed that gender (male), depth of tumor invasion (T4), extent of lymph node dissection (D0-2), number of examined lymph nodes (< 12), and POAC were significant independent prognostic factors for DFS. Clinical guidelines published by ASCO [2] and ESMO [4] also mention the depth of tumor invasion (T4) and number of examined lymph nodes (< 12) as risk factors for recurrence. A poorly differentiated component and a mucinous component were not risk factors for DFS and OS in the current study. This may be explained by the cases with these components having significantly lower rates of T4 invasion depth and examined lymph nodes < 12 (data not shown). In a large cohort in the National Cancer Database, male sex was found to be an independent risk factor for recurrence in stage II CRC cases that did not receive adjuvant systemic therapy [23]. Cases with and without POAC were included in the current study, but male sex was still identified as a risk factor for DFS and OS in stage II colon cancer. In the current study, multivariate analyses revealed that the extent of lymph node dissection was a risk factor for the DFS and OS in stage II colon cancer, as was the number of examined lymph nodes. D3 lymph node dissection is recommended for T3 or T4 primary tumor in the Japanese guidelines [6], in contrast to the clinical guidelines in western countries [2–5]. In the current study, D0-2 lymph node dissection was performed in only 15% of patients. Although this was presumably due to perforation/penetration, D3 lymph node dissection seems to be ideal for improving the prognosis of patients with stage II colon cancer. POAC with UFT/LV was found to be useful to improve DFS and OS, and several studies in a large cohort in the National Cancer Database have also shown that POAC is associated with a survival benefit for patients with high-risk features [24, 25]. In these studies, the kinds of POAC may have varied. Therefore, the current study is significant in terms of the consistent regimen of oral UFT/LV for stage II colon cancer with high-risk features. In addition, since the incidence of adverse events was low, oral UFT/LV appears to be safe and well-tolerated as POAC [8].

CEA mRNA in peripheral blood ≥ 24 h after curative resection of CRC has been found to be significantly correlated with poor long-term survival [9–13]. However, there was no correlation between a CEA mRNA-positive status and DFS or OS in the current study. The CEA mRNA-positive rate was 23.4%, which was similar to that of 24.1% in our previous study of the correlation between recurrence and CEA mRNA status in stage II, in which we also found no significant correlation with stage II cases in a small cohort (n = 87) [9]. Thus, the current study in a large multicenter cohort confirmed the lack of significance of CEA mRNA as a prognostic factor in stage II colon cancer. Therefore, measurement of CEA mRNA may not be beneficial for prediction of recurrence in stage II colon cancer. Circulating tumor DNA (ctDNA) has recently been reported to be a powerful tool to help guide treatment decisions in colorectal cancer. In patients with high-risk stage II or stage III disease, Kotani et al. [26] found that those with a ctDNA-positive status 4 weeks after surgery derived significant benefit from postoperative adjuvant chemotherapy, while those with a ctDNA-negative status 4 weeks after surgery did not. It was suggested that ctDNA positivity after curative surgery or therapy, which indicated minimal residual disease (MRD), was strongly associated with a poor prognosis in patients with surgically resectable CRC. Therefore, postsurgical ctDNA status is likely to be a future predictor of a benefit of postoperative adjuvant chemotherapy, instead of CEA mRNA.

Finally, there are several limitations in the current study that are inherent to the non-randomized design. First, the decision regarding the use of POAC with UFT/LV was made for each case based on individual patient/physician discussions. Second, some risk factors, such as MSI, were not included, but MSI status influences the benefit of POAC with fluoropyrimidine alone in colon cancer [27]. We also excluded lymphatic, vascular, or perineural invasion as a risk factor because its evaluation was likely to be inconsistent among the participating institutions. In fact, discrepancies in the observed rates in lymphovascular invasion in colorectal cancer have been widely reported, with Davenport et al. [28] suggesting that interobserver agreement on lymphovascular invasion is poorer than that for other clinicopathological factors.

## Conclusions

This study showed that gender (male), depth of tumor invasion (T4), extent of lymph node dissection (D0-2), number of examined lymph nodes (< 12), and lack of use of POAC were significant independent prognostic factors in stage II colon cancer.

## Data Availability

The datasets generated and/or analyzed during the current study are available from the corresponding author on reasonable request.
